# Chloroquine and Hydroxychloroquine, as Proteasome Inhibitors, Upregulate the Expression and Activity of Organic Anion Transporter 3

**DOI:** 10.3390/pharmaceutics15061725

**Published:** 2023-06-14

**Authors:** Zhengxuan Liang, Guofeng You

**Affiliations:** Department of Pharmaceutics, Rutgers, The State University of New Jersey, 160 Frelinghuysen Road, Piscataway, NJ 08854, USA

**Keywords:** organic transporter 3, proteasome inhibition, chloroquine, hydroxychloroquine, regulation

## Abstract

Organic anion transporter 3 (OAT3), at the basolateral membrane of kidney proximal tubule cells, facilitates the elimination of numerous widely used drugs. Earlier investigation from our laboratory revealed that ubiquitin conjugation to OAT3 leads to OAT3 internalization from the cell surface, followed by degradation in the proteasome. In the current study, we examined the roles of chloroquine (CQ) and hydroxychloroquine (HCQ), two well-known anti-malarial drugs, in their action as proteasome inhibitors and their effects on OAT3 ubiquitination, expression, and function. We showed that in cells treated with CQ and HCQ, the ubiquitinated OAT3 was considerably enhanced, which correlated well with a decrease in 20S proteasome activity. Furthermore, in CQ- and HCQ-treated cells, OAT3 expression and OAT3-mediated transport of estrone sulfate, a prototypical substrate, were significantly increased. Such increases in OAT3 expression and transport activity were accompanied by an increase in the maximum transport velocity and a decrease in the degradation rate of the transporter. In conclusion, this study unveiled a novel role of CQ and HCQ in enhancing OAT3 expression and transport activity by preventing the degradation of ubiquitinated OAT3 in proteasomes.

## 1. Introduction

Numerous widely used drugs, such as antiviral drugs, anticancer therapeutics, antibiotics, antihypertensives, and anti-inflammation drugs, are removed from the body through the organic anion transporter 3 (OAT3) at the basolateral membrane of the kidney proximal tubule cells [1,2,3]. OAT3 actively moves drugs from blood into tubule cells. Once inside the cells, these drugs are then pumped out of the apical membrane into urine by other carriers. Therefore, the activity of OAT3 is the deciding factor in the rate of renal clearance of substrate drugs from the body and affects their therapeutic effectiveness [4,5,6,7].

OAT3 transport activity vitally relies on its expression level at the plasma membrane, which can be changed under various diseased and pharmacological situations [1,8]. Our laboratory earlier demonstrated that OAT3 constitutively internalizes from and recycles back to the cell surface, and ubiquitin (an 8-kDa polypeptide) conjugation to OAT3 serves as a triggering signal for its internalization to early endosomes [9,10]. Prolonged ubiquitination leads the internalized OAT3 to target the proteasome for degradation [11,12].

Chloroquine, a well-known anti-malarial drug, was previously reported to have an inhibitory effect on proteasome in MDAY-D2 cell (a murine leukemia cell) extracts under supra-pharmacological concentrations [13]. However, its effect on proteasome-regulated OAT3 ubiquitination, expression, and transport function has never been explored. HCQ, a newer derivative of CQ and an anti-malarial drug, shows less toxicity, fewer side effects, and a better dissolution profile in the body. It is, therefore, often considered a safer medication for patients [14,15,16,17,18].

HCQ differs from CQ in its chemical structure by having a hydroxyl group at the end of the N-ethyl side chain [19]. Although often mentioned together, both drugs shine for their highlights. Besides the anti-malaria effect, CQ is also used to treat amebiasis [20]. HCQ has been widely used, since 2000, in the treatment of immune-mediated rheumatic disorders and has become a part of current treatment guidelines for rheumatoid arthritis, systemic lupus erythematosus, and antiphospholipid syndrome [21]. Furthermore, CQ and HCQ are also grabbing public attention through their values in cancer therapy, such as in solid cancers [22,23,24,25], pancreatic cancer [26], multiple myeloma [27], non-small cell lung cancer [28], and glioblastoma multiforme [29].

Both CQ and HCQ are predominantly renal eliminated with long half-lives, ranging from 20–60 days and 20–40 days [30,31], respectively, indicating their potential effects on renal function, and some studies also reported CQ’s effects on kidney both in rats and in human [32,33]. CQ and HCQ are weak bases at physiological pH due to the presence of a basic side chain [19,34]. This suggests that they are not substrates of OAT3. In the current study, we examined the roles of CQ and HCQ in their action as proteasome inhibitors and their effects on OAT3 ubiquitination, expression, and function.

## 2. Materials and Methods

### 2.1. Materials

Parental COS-7 cells and HEK293 cells were purchased from ATCC (Manassas, VA, USA). Hydroxychloroquine Sulfate and Chloroquine Disulfate were purchased from Sigma-Aldrich (St. Louis, MO, USA). Membrane-impermeable biotinylation reagent sulfo-NHS-SS-biotin, streptavidin agarose, protein G-agarose beads, and horseradish peroxidase-conjugated anti-mouse antibodies were obtained from Pierce Biotechnology (Rockford, IL, USA). We purchased [^3^H]-labeled estrone sulfate from PerkinElmer (Waltham, MA, USA). Mouse anti-myc antibody (9E10) was purchased from Roche (Indianapolis, IN, USA). Mouse anti-E-cadherin antibody was purchased from Abcam (Cambridge, MA, USA). Mouse anti-ubiquitin antibody, mouse anti-β-actin antibody and normal mouse IgG were purchased from Santa Cruz Biotechnology (Dallas, TX, USA). A 20S proteasome assay kit was purchased from Sigma-Aldrich (St. Louis, MO, USA). Normal mouse lgG, mouse monoclonal anti-ubiquitin and mouse monoclonal anti-β-actin antibodies were purchased from Santa Cruz (Santa Cruz, CA, USA).

### 2.2. Cell Culture and Transfection

Parental monkey kidney COS-7 cells and parental human embryonic kidney HEK293 cells were cultured in Dulbecco’s modified Eagle’s medium (DMEM) containing 10% fetal bovine serum as previously described [35]. Epitope myc was tagged to hOAT3 cDNA, and the plasmid was stably expressed in COS-7 cells and HEK293 cells. These cells were maintained in a DMEM medium containing 0.2 mg/mL G418 and 10% fetal bovine serum. Lipofectamine 2000 was used for the transfection of plasmids according to the manufacturer’s instructions. Some 48 h after the plasmids transfection, cells were harvested for further studies.

### 2.3. Measurement of 20S Proteasome Activity

Proteasome activity was measured using a 20S proteasome assay kit according to the manufacturer’s protocol. After the designated treatment, 100 mL/well of Proteasome Assay Loading Solution was added, and the 96-well plate was then incubated at 37 °C for 2 h, protected from light. The fluorescence intensity at λex = 490 nm and λem = 525 nm. was measured using a microplate reader (Tecan Infinite^®^ 200 PRO, Tecan, Morrisville, NC, USA).

### 2.4. Ubiquitination Assay

Nedd4-2, a ubiquitin ligase, was transfected into OAT3-myc-expressing cells to improve the basal OAT3 ubiquitination signal. Cells were lysed with lysis buffer (20 mM Tris/HCl, pH 7.5, 1% Triton X-100, 2 mM EDTA, and 25 mM NaF) freshly added with 1% of proteinase inhibitor cocktail and 20 mM N-ethylmaleimide (NEM) and then precleared with protein G agarose at 4 °C for 3 h to reduce nonspecific binding. Anti-myc antibody was incubated with protein G agarose at 4 °C for 4 h. The precleared protein sample was then mixed with antibody-bound protein G agarose and underwent end-over-end rotation at 4 °C overnight. Proteins bound to the beads were released and denatured by 2x Laemmle buffer (ThermoFisher Scientific, Waltham, MA, USA), followed by gel electrophoresis and immunoblotting.

### 2.5. Measurement of Transport Activity

Uptake of [^3^H]-estrone sulfate (300 nM) was performed, following the standard protocol previously established in our lab [36]. A 4-min-uptake study was carried out with OAT3-expressing COS 7 cells or OAT3-expressing HEK293 cells. A Beckman LSC LS6500 liquid scintillation counter was used for the measurement of [^3^H]-estrone sulfate in the cells. Passive diffusion data were collected with mock cells (parental COS-7 cells or parental HEK293 cells).

### 2.6. Kinetics of Estrone Sulfate Transport

OAT3-expressing cells were pretreated with or without 10 µM HCQ for 4 h, and 3-min uptake of [^3^H]-estrone sulfate was measured with an estrone sulfate concentration range of 0.3–10 µM. The same uptake procedure was applied as described in the section “Measurement of transport activity”. The kinetic values of estrone sulfate transported by OAT3 were determined using non-linear least-squares regression analysis from Michaelis–Menten equation: V = Vmax × [S]/(Km + [S]). Transport kinetic parameters were determined using the Eadie–Hofstee transformation.

### 2.7. Cell Surface Biotinylation

OAT3 at the cell surface was isolated and detected using the biotinylation method as described in our previous publication [37], followed by electrophoresis and immunoblotting using an anti-myc antibody.

### 2.8. Degradation Assay

OAT3-expressing cells were first labeled with biotinylation reagent sulfo-NHS-SS-biotin, then incubated with or without HCQ at 37 °C. The cells were collected at 0, 2, 4, and 6 h and lysed. The cell lysates were centrifuged, and the supernatant was then incubated with streptavidin agarose resin to isolate cell membrane proteins, followed by electrophoresis and immunoblotting.

### 2.9. Gel Electrophoresis and Immunoblotting

The electrophoresis and immunoblotting were performed using the method established in our lab [35]. Protein samples were resolved on 7.5% SDS-PAGE mini gels and electroblotted onto polyvinylidene difluoride membranes. The blots were blocked for 2 h with 5% nonfat dry milk in PBS-0.1% Tween 20 and incubated overnight at 4 °C with appropriate primary antibodies, followed by horseradish peroxidase-conjugated secondary antibodies. The SuperSignal West Dura Extended Duration Substrate kit was used to detect the signals. Nonsaturating immunoreactive protein bands were quantified by scanning densitometry with the FluorChem 8000 imaging system (Alpha Innotech Corp., San Leandro, CA, USA).

### 2.10. Data Analysis

Each experiment was repeated a minimum of three times. The statistical analysis was based on multiple experiments. Statistical analysis was performed using the Student’s paired t-tests between two groups. A *p*-value of <0.05 was considered statistically significant. The notation “ns” means “not statistically significant”.

## 3. Results

### 3.1. Effect of Chloroquine (CQ) and Hydroxychloroquine (HCQ) on 20S Proteasome Activity in OAT3-Expressing Cells

**Figure 1:**
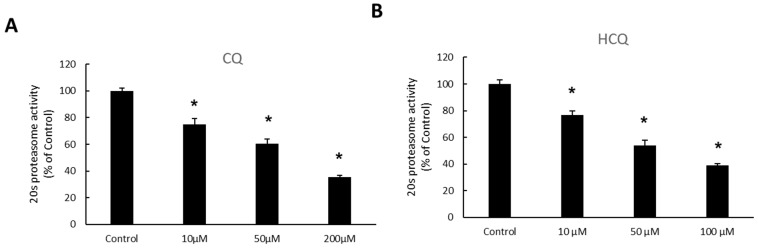
Effect of CQ (**A**) and HCQ (**B**) on 20S proteasome activity. COS-7 cells expressing OAT3 were incubated with CQ or HCQ at 10 μM for 4 h, followed by the measurement of the 20S proteasome activity. The 20S proteasome activity was expressed as the % of control cells from three independent experiments. Values are means ± S.D., (*n* = 3), and * *p* < 0.05.

As mentioned above, CQ was previously reported to have an inhibitory effect on proteasome in MDAY-D2 cell (a murine leukemia cell) extracts. However, the impacts of CQ and HCQ on proteasome-regulated OAT3 ubiquitination, expression, and transport function have never been explored. Therefore, we examined the effects of CQ and HCQ on the proteasome activity in OAT3-expressing cells of kidney origin. Our results showed that both CQ and HCQ significantly inhibited 20S proteasome activity (Figure 1). Treatment with CQ from 10–200 µM for 4 h led to the inhibition of the proteasome activity by 25–64%. Treatment with HCQ from 10–100 µM for 4 h led to the inhibition of the proteasome activity by 23–61%.

### 3.2. Effect of CQ and HCQ on OAT3 Ubiquitination

**Figure 2:**
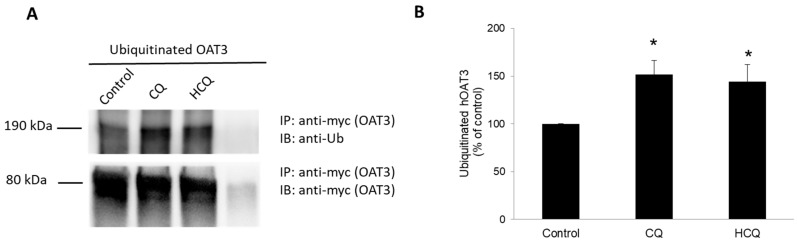
Effect of CQ and HCQ on OAT3 ubiquitination. (**A**) Top panel: OAT3-expressing COS-7 cells were treated with CQ or HCQ for 4 h at 10 µM. Treated cells were then lysed, and OAT3 was immunoprecipitated (IP) with an anti-myc antibody (OAT3 was tagged with myc) or mouse IgG (as negative control), followed by immunoblotting (IB) with anti-ubiquitin (Ub) antibody. Bottom panel: the same immunoblot from the top panel was reprobed with an anti-myc antibody to determine the amount of OAT3 immunoprecipitated. (**B**) Densitometry plot of results from (**A**) as well as from other repeat experiments. Values are means ± S.D., (*n* = 4), and * *p* < 0.05.

Our lab previously demonstrated that prolonged ubiquitination of OAT3 led the transporter to target the proteasome for degradation [11]. In this experiment, we examined the effect of CQ and HCQ on OAT3 ubiquitination (Figure 2). OAT3-expressing cells were treated with CQ or HCQ for 4 h. OAT3 was then immunoprecipitated from the treated cells by an anti-myc antibody (epitope myc was tagged to OAT3 for immunodetection), followed by immunoblotting (IB) with anti-ubiquitin (anti-Ub) antibody to detect ubiquitinated OAT3. As shown in Figure 2A, in the top panel, CQ and HCQ markedly increased the accumulation of ubiquitinated OAT3. The difference in ubiquitinated OAT3 did not arise from the amount of OAT3 pulled down since the amount of OAT3 immunoprecipitated was comparable among all samples (Figure 2A, bottom panel). The accumulation of ubiquitinated OAT3 with CQ or HCQ treatment was correlated with the inhibition of proteasome activity.

### 3.3. Time-Dependent Effect of CQ and HCQ on OAT3-Mediated Uptake of Estrone Sulfate

**Figure 3:**
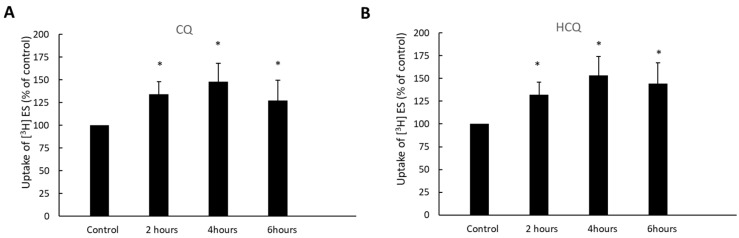
Effect of CQ (**A**) and HCQ (**B**) on OAT3 activity in a time-dependent manner. OAT3-expressing COS-7 cells were treated with 10 µM CQ or HCQ at indicated treatment time. The 4-min uptake of [^3^H] estrone sulfate (ES, 250 nM) was then carried out. Each data point reflected only carrier-mediated transport by subtracting values from parental cells. Uptake activity was expressed as the % of uptake measured in control cells from six independent experiments. Values are means ± S.D., (*n* = 6), and * *p* < 0.05.

OAT3-expressing cells were treated with CQ and HCQ for various time periods (2–6 h), and OAT3-mediated uptake of [^3^H]-labeled estrone sulfate was then measured. During our treatment, the fetal bovine serum was not included in the medium. Therefore, to maintain the cells in a healthy state, treatment longer than 6 h was not performed. The results (Figure 3) demonstrate that both CQ and HCQ induced stimulation of estrone sulfate uptake by ~30% at 2 h of treatment and by ~50% at 4 h of treatment. There was no further stimulation after 6 h of treatment. Therefore, the treatment time of 4 h was applied for all studies described in this paper.

### 3.4. Dose-Dependent Effect of CQ and HCQ on OAT3-Mediated Uptake of Estrone Sulfate

**Figure 4:**
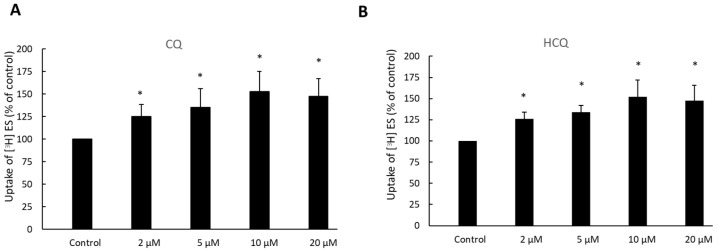
Effect of CQ (**A**) and HCQ (**B**) on OAT3 activity in a dose-dependent manner. OAT3-expressing COS-7 cells were treated with CQ or HCQ at the concentrations indicated for 4 h. A 4-min uptake of [^3^H] estrone sulfate (ES, 250 nM) was then carried out. Each data point reflected only carrier-mediated transport by subtracting values from parental cells. Uptake activity was expressed as the % of uptake measured in control cells from six independent experiments. Values are means ± S.D., (*n* = 6), and * *p* < 0.05.

OAT3-expressing cells were treated with CQ and HCQ for various concentrations, and OAT3-mediated uptake of [^3^H]-estrone sulfate was then determined (Figure 4). With 2 to 10 µM treatment, both CQ and HCQ induced stimulation of estrone sulfate uptake from ~25% to ~55%. There was no further stimulation at 20 µM of treatment. Therefore, the treatment dose of 10 µM was applied for all studies described in this paper.

### 3.5. Effect of CQ and HCQ on OAT3-Mediated Uptake of Estrone Sulfate in HEK293 Cells

**Figure 5:**
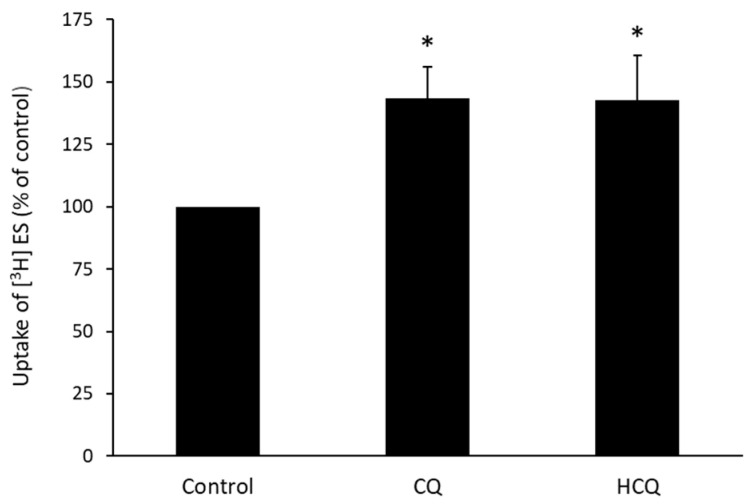
Effect of CQ and HCQ on OAT3 activity in HEK 293 cells. OAT3-expressing HEK293 cells were treated with CQ or HCQ at 10 μM for 4 h. A 4-min uptake of [^3^H] estrone sulfate (ES, 250 nM) was then carried out. Each data point reflected only carrier-mediated transport by subtracting values from parental cells. Uptake activity was expressed as the % of uptake measured in control cells from six independent experiments. Values are means ± S.D., (*n* = 6), and * *p* < 0.05.

The above experiments were carried out in COS-7 cells of monkey kidney origin. In this experiment, we examined the effect of CQ and HCQ on OAT3-mediated uptake of estrone sulfate in HEK293 cells of human kidney origin. Our result (Figure 5) showed that with the treatment concentration of 10 µM and treatment time of 4 h, compared to untreated cells, ~40% induction in uptake was observed in CQ- or HCQ-treated OAT3-expressing HEK293 cells, suggesting that stimulation of OAT3 activity by CQ and HCQ is not cell type-specific, but rather a general feature.

### 3.6. Kinetic Analysis of the Effect of HCQ on OAT3 Transport Activity

**Figure 6:**
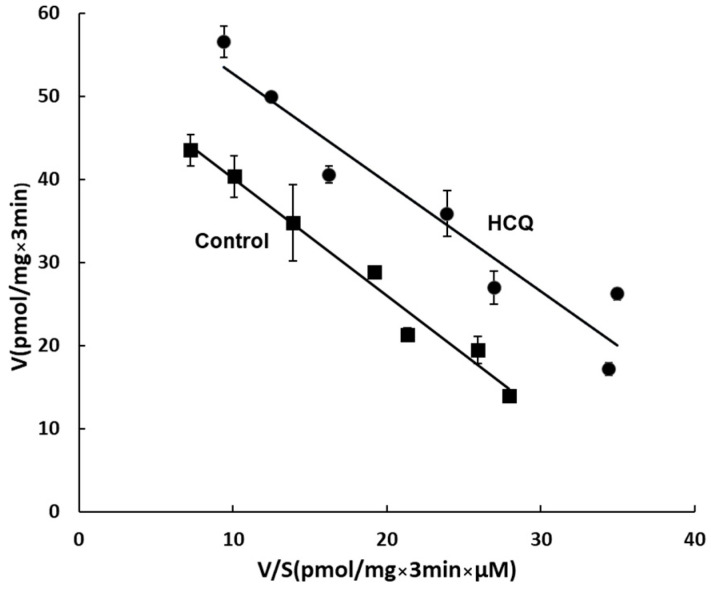
Effect of HCQ on the kinetics of OAT3-mediated estrone sulfate transport. OAT3-expressing COS-7 cells were treated with 10 μM HCQ for 4 h, and initial uptake (3 min) of [^3^H] estrone sulfate was measured at the concentration of 0.3~10 µM. The data represent uptake into OAT3-expressing cells minus uptake into mock cells (parental COS-7 cells). Values are means ± S.D., (*n* = 3), V = velocity, and S = substrate concentration.

To explore the mechanism underlying CQ- and HCQ-stimulated OAT3 activity, a kinetic analysis was carried out, with HCQ as the representative drug. We measured [^3^H]-estrone sulfate uptake at various substrate concentrations from 0.3 to 10 µM. Eadie–Hofstee analysis was applied for the data fitting [38]. As shown in Figure 6, with HCQ treatment, a substantial rise in maximum transport velocity (Vmax) was observed (65.8 ± 1.3 pmol·mg^−1^·3 min^−1^ with HCQ-treated cells and 54.1 ± 1.7 pmol·mg^−1^·3 min^−1^ with untreated cells), without notable alteration in the substrate-binding affinity Km.

### 3.7. Effect of CQ and HCQ on OAT3 Expression

**Figure 7:**
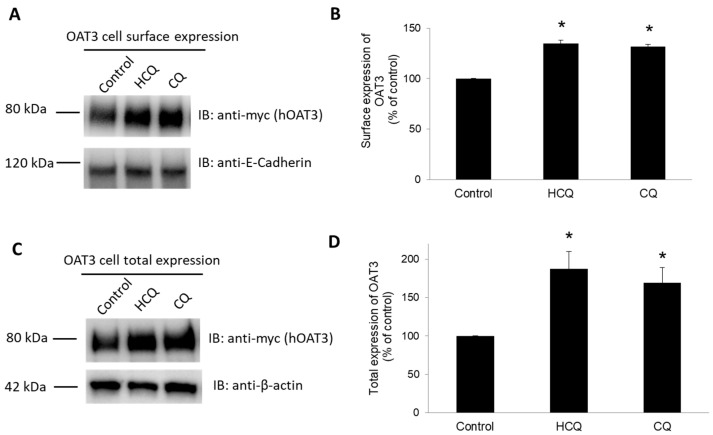
Effect of CQ and HCQ on OAT3 expression. (**A**) Top panel: OAT3-expressing COS-7 cells were treated with CQ or HCQ (10 µM, 4 h). Cell surface biotinylation was performed. Biotinylated (cell surface) proteins were separated using streptavidin agarose resin and analyzed by immunoblotting (IB) with an anti-myc antibody (OAT3 was tagged with myc). Bottom panel: the same blot from the top panel was reprobed with an antibody against a membrane protein marker E-Cadherin. (**B**) Densitometry plot of results from (**A**) and other repeat experiments. Values are means ± S.D., (*n* = 3)., and * *p* < 0.05. (**C**) Top panel: COS-7 cells expressing OAT3 were treated with CQ or HCQ (10 µM, 4 h), then lysed and followed by IB with anti-myc antibody. Bottom panel: the same blot from the top panel was reprobed with an antibody against a cellular protein marker β-actin. (**D**) Densitometry plot of results from (**C**) and other repeat experiments. Values are means ± S.D., (*n* = 3), and * *p* < 0.05.

OAT3-expressing cells were treated with CQ or HCQ, and OAT3 expression both at the cell surface and in the total cell lysates was analyzed (Figure 7). We showed that treatment with CQ and HCQ resulted in an enhancement of OAT3 expression at the cell surface (Figure 7A, top panel, and Figure 7B) and in total cell lysate (Figure 7C, top panel, Figure 7D). Such enhancement did not come from the overall disturbance of cellular proteins, as the expression of cell surface protein marker E-Cadherin (Figure 7A, bottom panel) and cellular protein marker β-actin (Figure 7C, bottom panel) remained unaffected under these circumstances.

### 3.8. Effect of HCQ on OAT3 Stability

**Figure 8:**
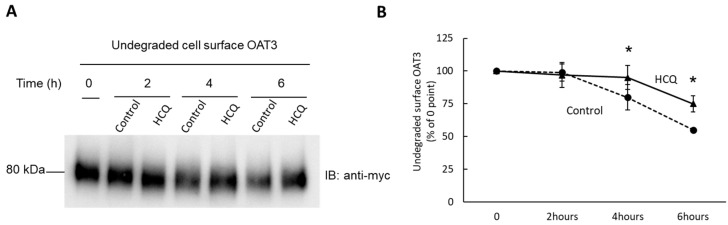
Effect of HCQ on the rate of OAT3 degradation. (**A**) OAT3 degradation (0, 2, 4, and 6 h) was analyzed as described under “Materials and Methods” with the treatment of 10 μM HCQ, followed by immunoblotting (IB) using anti-myc antibody (epitope myc was tagged to OAT3). (**B**) Densitometry plot of results from (**A**), as well as from other repeat experiments. The expression level was expressed as a percentage of cell surface OAT3 expression at 0 h. Statistical analysis was performed using the Student’s paired T-tests. Values are means ± S.E, (*n* = 3), and * *p* < 0.05.

We then determined the degradation rate of cell surface OAT3 using a biotinylation approach. COS-7 Cells expressing OAT3 were biotinylated with membrane-impermeable biotinylation reagent sulfo-NHS-SS-biotin. Labeled cells were treated with or without HCQ at 10 µM for 0, 2, 4, and 6 h and then lysed. Cell surface proteins were isolated by streptavidin-agarose resin, followed by immunoblotting with an anti-myc antibody. Our results (Figure 8) showed that from the fourth hour and onwards, the rate of OAT3 degradation reduced markedly with the treatment of HCQ in comparison with that of the control. These results indicate that HCQ markedly increased OAT3 stability.

## 4. Discussion

OAT3 transport activity critically relies on its expression level at the plasma membrane, which is subjected to various types of regulation [39,40,41]. We previously demonstrated that ubiquitination of OAT3 led to the internalization of the transporter from the plasma membrane to the intracellular endosome and subsequently targeted to the proteasome for degradation [9,42]. In the current study, we discovered a novel role of CQ and HCQ in stimulating OAT3 function by inhibiting proteasome activity and thereby preventing OAT3 degradation.

OAT3 recognizes a wide range of substrates [43]. As a result, drug–drug interaction can happen at the transporter molecule [44,45]. Simultaneous use of different drugs is common in combination therapy for treating single or multiple diseases. If one drug is an inhibitor, substrate, or inducer of OAT3, it will inhibit noncompetitively or competitively or stimulate OAT3-mediated transport of other drug substrates, causing potential drug–drug interactions (DDIs) [46,47]. In contrast to direct DDI, in which multiple drugs act directly on the transporter molecule, indirect DDI occurs when one drug acts on the regulatory machinery/pathway of the transporter instead of the transporter itself. This could lead to the alteration of transporter expression and function [1,48,49]. As we mentioned before, CQ and HCQ are not OAT3 substrates. Therefore, the increased OAT3 function with CQ or HCQ treatment is mainly due to their regulatory effects by proteasome inhibition. Consequently, CQ and HCQ can potentially cause DDIs in an indirect manner, affecting the safety and efficacy of many commonly used drugs that are OAT3 substrates.

Pharmacokinetically, both CQ and HCQ have long mean residence times caused by their potential to distribute in aqueous cellular and intercellular compartments (~50 days for hydroxychloroquine and ~40 days for chloroquine) [50,51,52]. Significant amounts of CQ and HCQ are eliminated in the kidney. Their plasma concentrations vary individually, ranging from 650–1300 ng/mL (2.0–4.1 µM) for CQ and 1161–2436 ng/mL (3.5–7.3 µM) for HCQ [30,53]. Therefore, the concentrations used in the current study for both drugs are within the clinically therapeutic range, which gives us a better clinical insight into the response of the patients taking CQ or HCQ.

The COS-7 cells and HEK293 cells used in the current study have several advantages: these cells are of kidney origin [54,55,56]. The lack of endogenous OATs allows the characterization of OAT3 without disturbance by other OATs. More importantly, the regulation of OAT3 in these cells exhibits similar mechanisms as that in vivo [57,58]. Therefore, the study in these cells paved the way for in vivo validation of the role of CQ and HCQ in OAT3 regulation.

CQ and HCQ markedly enhanced the accumulation of ubiquitinated OAT3 (Figure 2), which correlated well with a decrease in 20S proteasomal activity (Figure 1), upregulated OAT3-mediated transport of estrone sulfate (Figure 3, Figure 4 and Figure 5), and increased OAT3 expression (Figure 7). The augmented transport activity of OAT3 following drug pretreatment arose from an increase in maximum transport velocity (Figure 6) and a decrease in the rate of transporter degradation (Figure 8).

Previously several reports indicated that both CQ and HCQ have inhibitory effects on lysosomes [59,60,61,62,63,64,65]. Although lysosome plays an important role in the degradation of other membrane proteins, our recent publication demonstrated that ubiquitinated-OAT3 at the cell surface degraded only through proteasome but not lysosome [11]. Therefore, we focused on the effects of CQ and HCQ on the proteasome, not the lysosome. The only report about the inhibitory effect of CQ on proteasome was reported in MDAY-D2 cell (a murine leukemia cell) extracts under supra-pharmacological concentrations [13]. Our current study presented the first evidence for proteasome inhibition by HCQ and CQ in normal mammalian cells under physiology-relevant conditions.

Our discovery of CQ and HCQ in upregulating the activity and expression of OAT3 has significant physiological implications. First, with the OAT3 function of transporting endogenous substrate, CQ and HCQ can stimulate the renal clearance of metabolites, nutrients, signaling molecules, and other OAT3 substrates, which may disturb homeostasis. Additionally, CQ and HCQ stimulate the OAT3 transport activity, leading to increased elimination of the drugs that are OAT3 substances, thereby altering their efficacy. Moreover, as proteasome inhibitors, CQ and HCQ may have potential therapeutic effects on diseases in which the proteasome activity is abnormally high such as multiple myeloma, Crohn’s disease, and ulcerative colitis [66,67,68].

## 5. Conclusions

**Figure 9:**
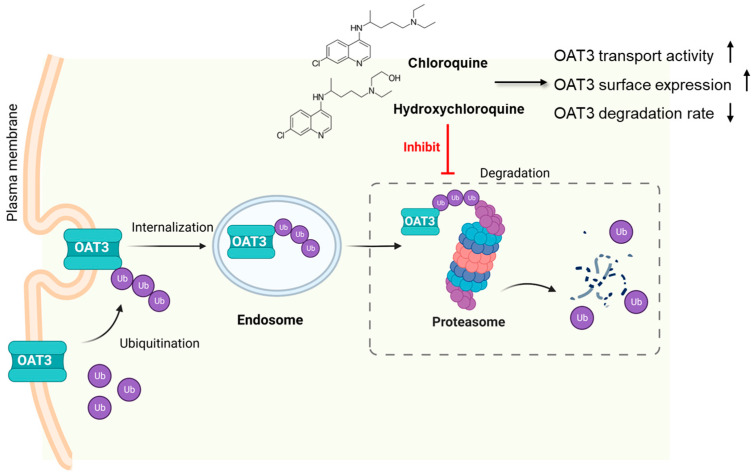
Chloroquine and Hydroxychloroquine upregulate the expression and transport activity of OAT3 by inhibiting proteasome.

Our current studies unveiled a novel role of both CQ and HCQ in upregulating OAT3 expression and function by inhibiting proteasome activity, thereby preventing the degradation of the transporter (Figure 9), which suggests their likely impact on the OAT3-mediated drug clearance and clinical drug–drug interactions during combination therapies using CQ/HCQ and other types of drugs.

## Data Availability

Not applicable.
